# COVID-19 Seroprevalence among Healthcare Workers of a Large COVID-19 Hospital in Rome Reveals Strengths and Limits of Two Different Serological Tests

**DOI:** 10.3390/ijerph18052650

**Published:** 2021-03-06

**Authors:** Giuseppe Vetrugno, Daniele Ignazio La Milia, Floriana D’Ambrosio, Marcello Di Pumpo, Roberta Pastorino, Stefania Boccia, Rosalba Ricci, Fabio De-Giorgio, Michela Cicconi, Federica Foti, Domenico Pascucci, Francesco Castrini, Elettra Carini, Andrea Cambieri, Maria Elena D’Alfonso, Gennaro Capalbo, Massimo Fantoni, Umberto Moscato, Domenico Staiti, Francesco Maria De Simone, Filippo Berloco, Gianfranco Damiani, Maurizio Zega, Paola Cattani, Brunella Posteraro, Maurizio Sanguinetti, Patrizia Laurenti

**Affiliations:** 1 Risk Management Unit, Fondazione Policlinico Universitario A. Gemelli IRCCS, 00168 Rome, Italy; giuseppe.vetrugno@policlinicogemelli.it (G.V.); michelacicconi@gmail.com (M.C.); fotifederica89@gmail.com (F.F.); 2 Section of Legal Medicine, Department of Health Care Surveillance and Bioethics, Università Cattolica del Sacro Cuore, 00168 Rome, Italy; fabiodegiorgio3@gmail.com; 3 Hospital Health Management, Fondazione Policlinico Universitario A. Gemelli IRCCS, 00168 Rome, Italy; danieleignazio.lamilia@policlinicogemelli.it (D.I.L.M.); andrea.cambieri@policlinicogemelli.it (A.C.); mariaelena.dalfonso@policlinicogemelli.it (M.E.D.); gennaro.capalbo@unicatt.it (G.C.); filippo.berloco@policlinicogemelli.it (F.B.); 4 Section of Hygiene, University Department of Health Sciences and Public Health, Università Cattolica del Sacro Cuore, 00168 Rome, Italy; dipumpomarcello@gmail.com (M.D.P.); Stefania.boccia@unicatt.it (S.B.); domenico.pascucci@outlook.it (D.P.); fr.castrini@gmail.com (F.C.); elettra.carini1@gmail.com (E.C.); Umberto.moscato@policlinicogemelli.it (U.M.); gianfranco.damiani@unicatt.it (G.D.); patrizia.laurenti@policlinicogemelli.it (P.L.); 5 Department of Woman and Child Health and Public Health—Public Health Area, Fondazione Policlinico Universitario A. Gemelli IRCCS, 00168 Rome, Italy; Roberta.pastorino@policlinicogemelli.it (R.P.); domenico.staiti@policlinicogemelli.it (D.S.); francescomaria.desimone@policlinicogemelli.it (F.M.D.S.); 6 Department of Laboratory and Infectivological Sciences, Fondazione Policlinico A. Gemelli IRCCS, 00168 Rome, Italy; rosalba.ricci@policlinicogemelli.it (R.R.); massimo.fantoni@policlinicogemelli.it (M.F.); paola.cattani@policlinicogemelli.it (P.C.); maurizio.sanguinetti@policlinicogemelli.it (M.S.); 7 Section of Infectious Diseases, Department of Health Care Surveillance and Bioethics, Università Cattolica del Sacro Cuore, 00168 Rome, Italy; 8 Occupational Health Section, University Department of Health Sciences and Public Health, Università Cattolica del Sacro Cuore, 00168 Rome, Italy; 9 Director of Nursing Service Technician and Rehabilitation Administration (S.I.T.R.A.), Fondazione Policlinico Universitario Agostino Gemelli, 00168 Rome, Italy; Maurizio.zega@policlinicogemelli.it; 10 Department of Basic Biotechnological Sciences, Intensive and Perioperative Clinics, Università Cattolica del Sacro Cuore, 00168 Rome, Italy; brunella.posteraro@policlinicogemelli.it; 11 Department of Medical and Surgical Sciences, Fondazione Policlinico Universitario A. Gemelli IRCCS, 00168 Rome, Italy

**Keywords:** COVID-19, healthcare workers, point-of-care, SARS-CoV-2, serological tests, seroprevalence

## Abstract

Healthcare workers are at the forefront against COVID-19, worldwide. Since Fondazione Policlinico Universitario A. Gemelli (FPG) IRCCS was enlisted as a COVID-19 hospital, the healthcare workers deployed to COVID-19 wards were separated from those with limited/no exposure, whereas the administrative staff were designated to work from home. Between 4 June and 3 July 2020, an investigation was conducted to evaluate the seroprevalence of severe acute respiratory syndrome coronavirus 2 (SARS-CoV-2) immunoglobulin (IgG) antibodies among the employees of the FPG using point-of-care (POC) and venous blood tests. Sensitivity, specificity, and predictive values were determined with reverse-transcription polymerase chain reaction on nasal/oropharyngeal swabs as the diagnostic gold standard. The participants enrolled amounted to 4777. Seroprevalence was 3.66% using the POC test and 1.19% using the venous blood test, with a significant difference (*p* < 0.05). The POC test sensitivity and specificity were, respectively, 63.64% (95% confidence interval (CI): 62.20% to 65.04%) and 96.64% (95% CI: 96.05% to 97.13%), while those of the venous blood test were, respectively, 78.79% (95% CI: 77.58% to 79.94%) and 99.36% (95% CI: 99.07% to 99.55%). Among the low-risk populations, the POC test’s predictive values were 58.33% (positive) and 98.23% (negative), whereas those of the venous blood test were 92.86% (positive) and 98.53% (negative). According to our study, these serological tests cannot be a valid alternative to diagnose COVID-19 infection in progress.

## 1. Introduction

In December 2019, a cluster of unknown acute respiratory illnesses occurred in Wuhan city, Hubei province, China, and rapidly spread to other areas in the following months [1,2]. The responsible agent was identified by the Chinese Centre for Disease Control and Prevention (CCDC) on 7 January 2020 and was subsequently named severe acute respiratory syndrome coronavirus 2 (SARS-CoV-2). The disease was later named (COronaVIrus Disease 19 (COVID-19) by the World Health Organization (WHO) [3]. Due to the widespread global transmission of COVID-19 and the high rate of contagiousness, the WHO declared COVID-19 to be a pandemic on 11 March 2020 [3]. Early in the SARS-CoV-2 outbreak, several healthcare workers (HCWs) were infected while providing care to patients with COVID-19 [4,5,6]. Identification and isolation of infected and potentially infectious HCWs is indeed relevant to protect them and their families and may also prevent onward transmission to patients and colleagues, as well as reduce the risk of healthcare-associated outbreaks [5]. In Italy, COVID-19 cases increased rapidly from 23 February 2020, with SARS-CoV-2 spreading mostly in northern regions, particularly in the Lombardy region, where, on 4 June 2020, the number of COVID-19 cases was 89,526 (38.26% of the total cases in Italy). The heavy impact of COVID-19 was also highlighted by the estimation of its burden through Disability-adjusted life years (DALYs) computation. Indeed, by the end of April 2020, the total burden of COVID-19 in Italy was 121,449 DALYs [7]. Although, in the Lazio region, on the same date, the number of COVID-19 cases was 7764, the Fondazione Policlinico Universitario A. Gemelli (FPG) IRCCS (Istituti di Ricovero e Cura a Carattere Scientifico)—a large teaching university hospital in Rome that was enlisted as a COVID-19 hospital—had treated 553 COVID-19 patients, 133 of them in the intensive care unit (ICU). Based on this evidence and by agreement with the Lazio region, [8] the FPG launched a seroprevalence investigation to assess the potential contagion sources among FPG HCWs. Previous studies have already discussed data on HCWs’ seroprevalence across different countries worldwide, indicating that isolation protocols, hygiene standards, and personal protective equipment (PPE) may prevent high levels of nosocomial transmission [9,10,11,12,13,14,15,16,17,18].

The current diagnostic tests for COVID-19 fall into two main categories: molecular tests detecting SARS-CoV-2 RNA, and serological tests detecting anti-SARS-CoV-2 immunoglobulins (Igs; i.e., IgG/IgM) [19]. The reverse-transcription polymerase chain reaction (RT-PCR) molecular test, usually performed on nasal/oropharyngeal swab (NOS) samples, is considered the reference standard for COVID-19 diagnosis [20]. However, this test has long turnaround times (it takes over 2 to 3 h to generate results) and requires certified laboratories, expensive equipment, and trained technicians to operate. Limitations include potential false-negative results and precarious availability of test materials [19,20]. Conversely, serological tests have been proposed as an alternative to RT-PCR in cases of acute SARS-CoV-2 infection [21]. They are cheaper and easier to implement in laboratory diagnostics for SARS-CoV-2, especially in a point-of-care (POC) format. A clear advantage of these tests over RT-PCR is that they can identify individuals previously infected by SARS-CoV-2, even if they did not undergo testing while acutely ill [19]. Considering their short time of appearance from the onset of SARS-CoV-2 infection, viral-specific IgG and IgM antibodies could indicate an ongoing infection [20]. In this regard, population-based sero-epidemiological surveys, especially in healthcare settings, quantifying the proportion of individuals with anti-SARS-CoV-2 antibodies, may be very helpful [22]. The aim of this study was to assess the seroprevalence of SARS-CoV-2-specific IgG antibodies among HCWs of the FPG using serological tests, which rely on venous or capillary blood sampling. The sensitivity and specificity of both tests, namely, the venous blood and POC tests, were evaluated in comparison with RT-PCR results on NOS samples from FPG HCWs.

## 2. Materials and Methods

### 2.1. Study Participants and Design

This cross-sectional study consisted of a seroprevalence survey between 4 June 2020 and 3 July 2020, which enrolled participants on a voluntary basis via a hospital e-mail system, including medical, non-medical HCW, and administrative staff (AS) of the FPG (Table 1). The study was approved by the FPG ethics committee (ID number 3253) and participants signed an informed consent form before their inclusion in the study. Both the venous blood and POC SARS-CoV-2 serological tests were offered to each participant and performed in dedicated blood-drawing areas in compliance with COVID-19 safety regulations. In cases with a positive result from at least one test, participants underwent NOS sampling for RT-PCR SARS-CoV-2 RNA detection to assess the actual infection status [8]. Unlike the venous blood testing, the POC testing was performed by trained clinical staff composed of public health residents and student nurses.

Medical and non-medical HCWs were categorized into two groups by whether they had or had not assisted COVID-19 patients in the period between 9 March 2020 (the date of the first COVID-19 patients in our hospital) and 4 June 2020 (the date of seroprevalence survey initiation). For predictivity analysis, which requires the consideration of the prevalence of the studied population, we used the AS as a further comparison group, because this was a group with low seroprevalence. This is because these participants were less exposed to COVID-19 infection than HCWs and many of them had been in work-from-home arrangements for two days a week from 9 March 2020.

As mentioned above, participants that tested positive for SARS-CoV-2 specific antibodies, with at least one of the serological tests used in the study (see below), were sampled for NOS testing [23] within 48 h after positive serological test results were available. RT-PCR testing on NOS samples was performed using the Seegene Allplex™ 2019-nCoV assay, and a positive result (i.e., a Ct less than 40) for at least one of two viral targets (i.e., RdRP and N genes) indicated the presence of SARS-CoV-2 RNA. As current studies show marked variation and are likely to overestimate sensitivity, we used the lower end of current estimates from systematic reviews, with the approximate numbers of 70% for sensitivity and 95% for specificity, for illustrative purposes [24].

### 2.2. Detection of Anti-SARS-CoV-2 Antibodies in Blood Samples

Two serological tests were used to detect SARS-CoV-2-specific antibodies in participants’ blood samples. In the venous blood tests, samples were subjected to an enzyme-linked immunosorbent assay (ELISA) marketed by Euroimmun (Lübeck, Germany; www.euroimmun.com, last accessed on 2 January 2021) for SARS-CoV-2 IgG detection. Each kit contained microplate strips with 8 break-off reagent wells coated with S1 domain of viral spike protein recombinant of SARS-CoV-2 [25]. In the POC tests, capillary blood samples were directly subjected to the AllTestTM 2019-nCoV IgG/IgM Rapid Test Cassette assay lateral-flow chromatographic immunoassay for SARS-CoV-2 IgG/IgM detection [20,26].

### 2.3. Detection of COVID-19 Infection among Healthcare Workers

The collection of respiratory tract specimens, through NOS, to confirm COVID-19 status was routinely performed at least once on 5270 HCWs who met the following criteria: symptomatic participants; contact without adequate PPE with a COVID-19 case; HCWs employed in a ward with COVID-19 patients (swab performed monthly); HCWs employed in a ward with COVID-19 patients (swab performed bi-monthly); HCWs not employed in a COVID-19 ward (swab performed quarterly) [20].

### 2.4. Sensitivity and Specificity of Serological Tests

The sensitivity and specificity of the ELISA-based venous blood tests and the POC tests were assessed using RT-PCR assay as the diagnostic gold standard. We included only RT-PCR results from NOS samples obtained from the participants at least 30 days beforehand. This allowed the definition of the seroconversion time for each participant with a positive serological result. As a comparison group for predictive value analysis, we considered all participants enrolled in the study with at least one negative RT-PCR molecular test and only AS with at least one negative RT-PCR molecular test, because HCWs are more exposed to COVID-19 infection risk than the general population. The AS group, as with the general population, are likely to have a lower risk of positivity to SARS-CoV-2 than the most exposed groups, such as HCWs or quarantined persons who had been exposed to SARS-CoV-2 [27].

### 2.5. Statistical Analysis

Descriptive analysis was performed for sex, age, professional category, and wards of the HCWs. The difference between proportions was evaluated with the two proportion Z test. Seroprevalence was calculated, separately, for tests on the venous samples and the POC tests. Seroprevalence for the tests on the venous samples was estimated as the proportion of individuals who had a positive result of IgG in the immunoassay. Furthermore, for the tests on capillary blood, seroprevalence for both IgM and IgG was estimated as the proportion of individuals who had positive results in the corresponding band of the POC test.

We also re-estimated the sensitivity and specificity of the POC test using the immunoassay as a reference. The accuracy of the capillary versus the venous test was evaluated with sensitivity, specificity, and predictive values with 95% confidence intervals (CIs) [19]. Each of the seroprevalences were stratified for professional category, age, and wards in which they worked during the COVID-19 emergency.

The difference between positivity in one of the serological tests and positivity in the RT-PCR on NOS samples was estimated through Pearson’s chi-squared tests. The Spearman rank test (Bonferroni-adjusted) was used to evaluate the correlation between the anti-SARS-CoV-2 IgG assay in capillary blood versus the same titer in venous blood. Furthermore, Cronbach’s alpha was evaluated. In general, significant reliability values for Cronbach’s alpha are to be considered those >0.70.

Statistical analyses were carried out using software for the construction of the general basic dataset (Microsoft Excel for Mac Version 16.35), and specific software for the inferential statistical analysis (Stata Corp 4905 Lakeway, College Station, USA Stata/IC 14.2 for Mac (64-bit Intel), revision 29 January 2018).

## 3. Results

Of the 7889 eligible participants, 4888 (62%) responded to the invitation and 111 of these refused to participate. Therefore, 4777 participants were enrolled in the seroprevalence investigation. Of the enrolled participants, 295 participants expressed consent to the venous blood test only, 83 expressed consent to the POC test only, and 4399 expressed consent to both. Table 1 reports the general characteristics of the participants. Their mean age was 43.11 years (SD, ±11.51), while age data were missing for 122 participants. Females accounted for 65.35% (3122/4777) of the total; 31% (1481/4777) of the participants were nurses and 17.35% (829/4777) medical doctors. Around 15% (736/4777) of the participants were employed in COVID-19 wards.

The rate of positivity for SARS-CoV-2 RNA detected on NOS samples was 0.85% (45/5270). Conversely, POC seroprevalence was 3.66% (164/4482) and venous blood test seroprevalence was 1.19% (56/4694). Stratified results are shown in Table 2. The number of participants tested at least once for SARS-CoV-2 RT-PCR detection in NOS samples was 3538. Of these, 3184 had an RT-PCR result at least 30 days before serological testing and these results were considered in the accuracy analysis of the serological tests.

Considering all participants enrolled, the POC test showed a sensitivity of 63.64% (95% CI: 62.20% to 65.04%) and a specificity of 96.64% (95% CI: 96.05% to 97.13%), whereas the venous blood test showed a sensitivity of 78.79% (95% CI: 77.58% to 79.94%) and a specificity of 99.36% (95% CI: 99.07% to 99.55%). Conversely, considering only the AS comparison group, the POC test showed a sensitivity of 63.64% (95% CI: 59.10% to 67.93%) and a specificity of 97.79% (95% CI: 95.90% to 98.85%), whereas the venous blood test showed a sensitivity of 78.79% (95% CI: 74.89% to 82.22%) and a specificity of 99.57% (95% CI: 98.36% to 99.92%). Positive predictive values (PPVs) and negative predictive values (NPVs) for the POC and the venous blood tests, and stratification of PPVs, NPVs, sensitivity, and specificity with both the AS and the total participants as comparison groups are shown in Table 3.

The Pearson’s chi-squared test showed a significant difference between the POC and venous blood test results (*p* < 0.05).

Out of 4683 observations, the value of Spearman’s rho was 0.1052, with a *p*-value < 0.00.

Cronbach’s alpha showed a low concordance (scale reliability coefficient: 0.4477; *p* < 0.05).

Of the 7889 eligible participants, 45 HCWs were COVID-19 known cases before the beginning of the seroprevalence survey. Of these cases, 33 were enrolled in the survey and 26 of the 33 had positive results for SARS-CoV-2 IgG with the venous blood test. None of the participants with positive results from the venous blood or POC tests, and which were consequently tested for SARS-CoV-2 infection by RT-PCR, had detectable SARS-CoV-2 RNA in their NOS samples.

All individuals tested, both by venous blood test and POC test, were asymptomatic. Out of the HCWs that previously tested positive using qPCR, four were hospitalized. Among the four hospitalized cases, three tested positive for IgG antibodies, both for the venous test and the POC test. As a result, we did not perform any correlation tests because of the small size (i.e., four cases) of the population described above.

## 4. Discussion

Our results showed that the rate of SARS-CoV-2 infection and serological positivity in different work categories is consistent with the low spread of SARS-CoV-2 in the FPG, which differs from the national surveillance data that report a seroprevalence of 2.5% [28]. Furthermore, the seroprevalence for HCWs ranged between 1.6% and 14.6% in several studies [9,10,11,12,13,14,15,16,17,18].

We found a slight difference (but not statistically significant) in the seroprevalence determined by venous blood tests between HCWs who had or had not assisted in COVID-19 wards; this is in accordance with another study that reported a higher seroprevalence rate than the one reported in our study [9]. The highest seropositivity rates (by venous blood test) among the different worker categories were observed for medical doctors. Although this finding is not of immediate interpretation, a possible reason could be their exposure to high-risk procedures (i.e., oral intubation, reanimation, and clinical examination). Overall, we observed differences in seropositivity rates among age categories (statistically significant in both the 46–55 and >56 years age classes) whereas the above-mentioned differences were not statistically significant between sex categories.

Our study showed that the specificity was high for the venous blood test but not for the POC test. Conversely, using RT-PCR assay as the diagnostic gold standard, the venous blood test sensitivity might meet the criteria for screening tests, unlike the POC test’s sensitivity. NPVs were acceptable for both tests, whereas the PPVs were acceptable only for the venous blood test, as shown in Table 3. A systematic review and meta-analysis by Bastos et al. [19] revealed that the current evidence does not support continued use of existing POC tests for COVID-19 serology. On the one hand, our study showed high PPVs for venous blood serological testing among a low-risk population (i.e., AS) of hospital staff, while on the other hand, this finding may not be the case for a medium-/high-risk population (i.e., the entire working community of a COVID-19 hospital).

We also observed that, among the subset of participants who tested positive with the venous blood or POC test and who were consequently tested for SARS-CoV-2 infection by RT-PCR, none of these participants had detectable SARS-CoV-2 RNA in their NOS samples, thus confirming that the serological test is not useful to diagnose COVID-19 infection in progress. At the base of what is described above is that the PCR is considered worldwide as the gold standard over the serological test, which, on the contrary, is not widely accepted. Moreover, the clinical history of the considered population was already known, as detailed in the methods section. Furthermore, of the 33 COVID-19 cases already known and previously tested for SARS-CoV-2 IgG in the venous blood, only 26 (78.8%) of these tested positive, thus highlighting the disagreement in recent observations [27] and confirming uncertainties about infections that occurred more than five weeks before the tests [21,29]. Our evidence suggests a decrease of the antibody titer, which, in turn, could corroborate the hypothesis of a non-persistent immunity and ultimately justify a possible re-infection with SARS-CoV-2 [30]. Furthermore, our sample group, while small in size, was composed mainly of mild or asymptomatic cases (only two of 33 cases were admitted to the ICU). As proposed by Burgess et al. [31], the severity of illness is linked to the magnitude of serological responses. This association is also suggested by the experience gathered from other coronaviruses [32].

Our large teaching hospital gave us the opportunity to enroll a considerable sample size. Furthermore, even if some of the published studies involved larger numbers, our study is the first to stratify the sample by risk. From the beginning of the pandemic in Italy, the hospital directorate decided to distinguish HCWs working in dedicated COVID-19 wards from those with limited or no exposure (i.e., working in non-COVID-19 wards), and allowed AS to work from home. We assumed that the latter group has the same risk as the general population, as the AS were employed in work-from-home arrangements during the Italian lockdown period.

Our study has several limitations. The sample was not drawn randomly and the estimation of the seroprevalence was also subject to other potential sampling biases, due to the voluntarily enrolling procedure. Moreover, the sensitivity of the serological test could be biased because it depended on the test time from the onset of disease. Samples collected from infected individuals outside the time window of antibody response could produce false negatives and, therefore, the observed seroprevalence in our study could have potentially underestimated the true prevalence of the disease.

Another limitation of the study could be the COVID-19 case detection strategy. In fact, to ascertain the exact number of COVID-19 cases among HCWs, a systematic and periodic (ideally every 14 days) screening with RT-PCR from NOS samples should be performed. However, this was not sustainable during the emergency due to lack of resources.

Owing to the cross-sectional design of this study, the dynamic changes of the antibody titer in infected individuals over time were not evaluated.

Further studies, especially with long-term follow-up, will be needed in the future to assess the value of serological tests, considering their major public health implications.

## 5. Conclusions

Evidence in the scientific literature showed that POC tests have low diagnostic accuracy.

Taking into account a low-risk population, the capacity of the venous blood tests to identify the proportion of participants who tested positive seems to meet the criteria for screening tests, but it is not useful to diagnose COVID-19 past exposure.

According to our study, serological tests cannot be a valid alternative to diagnose COVID-19 infection in progress. However, considering the dynamic changes of antibodies to SARS-CoV-2 in COVID-19 patients, further studies are needed to highlight the best window of time for antibody response.

The discordance between the serological test and the RT-PCR molecular test among the confirmed COVID-19 cases could be explained by the limited sample size analyzed in this study. Further studies are needed to confirm this observation.

## Figures and Tables

**Table 1:** General characteristics of enrolled participants.

Participants’ Characteristics	*N* (%)
Age class *	
<36 years	1414 (30.38)
36–45 years	1127 (24.21)
46–55 years	1240 (26.64)
>56 years	874 (18.76)
Sex	
Males	1655 (34.65)
Females	3122 (65.35)
Professional category	
Medical doctors	829 (17.35)
Nurses	1481 (31.00)
Medical residents	552 (11.56)
Other HCWs	1059 (22.17)
Administrative staff	474 (9.92)
External workers	207 (4.33)
Others/unclassified	175 (3.66)
COVID-19 care	
Yes	736 (15.41)
No	4041 (84.96)
Total participants	4777 (100)

* For 122 individuals, information regarding age was missing. HCWs, healthcare workers.

**Table 2:** Prevalence of severe acute respiratory syndrome coronavirus 2 (SARS-CoV-2) infection based on the three tests and stratified by age, sex, professional category, and direct assistance to COVID-19 patients or not (COVID-19 care, Yes/No).

	Participants with a Positive Result for Each Test (%)		
Participants’ Characteristics	Venous Blood Test (%)	POC Test (%)	RT-PCR Test (%)
Overall	56 (1.19%)	164 (3.66%)	45 (0.85%)
Age class			
<36 years	16 (1.15%)	4 (3.75%)	16 (0.86%)
36–45 years	11 (1.62%)	47 (3.57%)	10 (0.91%)
46–55 years	8 (0.82%)	28 (2.96%) *	8 (0.62%)
>56 years	8 (1.40%)	43 (4.98%) *	10 (0.89%)
Sex			
Males	23 (0.01%)	45 (0.02%)	18 (0.01%)
Females	33 (0.01%)	119 (0.04%)	27 (0.01%)
Professional category			
Medical doctors	20 (2.43%) ^1^	30 (3.80%) ^3^	14 (1.34%)
Nurses	16 (1.10%) ^2^	59 (4.25%) ^4^	15 (0.77%)
Medical residents	4 (0.74%) ^2^	20 (3.74%) ^3^	6 (0.66%)
Other HCWs	11 (1.06%) ^2^	39 (3.93%) ^1^	9 (0.75%)
Administrative staff	2 (0.43%) ^2^	10 (2.21%) ^6^	0 (0.00%)
External workers	0 (0.00%) ^2^	1 (0.52%) ^5^	0 (0.00%)
Others/unclassified	3 (1.83%) ^2^	5 (3.82%) ^3^	1 (1.49%)
COVID-19 care			
Yes	10 (1.38%)	33 (4.98%) *	12 (1.10%)
No	46 (1.16%)	131 (3.43%)	33 (0.79%)

^1^ vs. nurses, medical residents, other HCWs, administrative staff, and external workers. ^2^ vs. medical doctors only. ^3^ vs. external workers only. ^4^ vs. external workers and administrative staff. ^5^ vs. all other categories. ^6^ vs. medical doctors, nurses, and external workers. * *p* < 0.05. POC, point-of-care.

**Table 3:** Diagnostic parameters for the POC and venous blood tests stratified with both the administrative staff (AS) and total participants as comparison groups.

	Administrative Comparison Group			Total Participants Comparison Group		
		Lower Limit	Upper Limit		Lower Limit	Upper Limit
Venous blood test						
Sensitivity	78.79%	74.90%	82.23%	78.79%	77.59%	70.95%
Specificity	99.57%	98.37%	99.92%	99.36%	99.07%	99.56%
Negative predictive value	98.53%	96.92%	99.34%	99.85%	99.68%	99.93%
Positive predictive value	92.86%	90.15%	94.88%	46.43%	44.99%	47.87%
POC test						
Sensitivity	63.64%	59.11%	67.94%	63.64%	62.21%	65.04%
Specificity	97.79%	95.91%	98.85%	96.64%	96.06%	97.13%
Positive predictive value	58.33%	53.74%	62.79%	8.54%	7.74%	9.40%
Negative predictive value	98.23%	96.46%	99.15%	99.81%	99.63%	99.92%

## Data Availability

All relevant data are within the manuscript and its Supporting Information files.
